# Development and Validation of an IMU Sensor-Based Behaviour-Alert Detection Collar for Assistance Dogs: A Proof-of-Concept Study

**DOI:** 10.3390/ani15213081

**Published:** 2025-10-23

**Authors:** Shelley Brady, Alan F. Smeaton, Hailin Song, Tomás Ward, Aoife Smeaton, Jennifer Dowler

**Affiliations:** 1 Insight Research Ireland Centre for Data Analytics, Dublin City University, D09 V209 Dublin, Ireland; alan.smeaton@dcu.ie (A.F.S.);; 2 Dogs for the Disabled, T12 E264 Cork, Ireland

**Keywords:** seizure-alert dogs, assistance animals, wearable sensors, machine learning, epilepsy monitoring, Internet of Medical Things (IoMT), Internet of Animals

## Abstract

**Simple Summary:**

Seizure-alert dogs can offer early warnings of seizures to individuals with epilepsy, yet existing approaches to using alert dogs rely on spontaneous behaviours that are difficult to validate or replicate. This study presents a wearable behaviour-alert detection collar designed to recognise signalling behaviours in trained assistance dogs using machine learning and motion sensors. Data were collected from six trained dogs performing a standardised spin alert behaviour, producing 135 labelled spin events. By standardising the alert behaviour and automating detection, the system achieved reliable recognition of spins across dogs, with cross-dog accuracy reaching up to 92.4%. This prototype demonstrates a novel, animal-integrated solution for improving seizure response and care.

**Abstract:**

Assistance dogs have shown promise in alerting to epileptic seizures in their owners, but current approaches often lack consistency, standardisation, and objective validation. This proof-of-concept study presents the development and initial validation of a wearable behaviour-alert detection collar developed for trained assistance dogs. It demonstrates the technical feasibility for automated detection of trained signalling behaviours. The collar integrates an inertial sensor and machine learning pipeline to detect a specific, trained alert behaviour of two rapid clockwise spins used by dogs to signal a seizure event. Data were collected from six trained dogs, resulting in 135 labelled spin alerts. Although the dataset size is limited compared to other machine learning applications, this reflects the real-world constraint that it is not practical for assistance dogs to perform excessive spin signalling during their training. Four supervised machine learning models (Random Forest, Logistic Regression, Naïve Bayes, and SVM) were evaluated on segmented accelerometer and gyroscope data. Random Forest achieved the highest performance (F1-score = 0.65; accuracy = 92%) under a Leave-One-DOG-Out (LODO) protocol. The system represents a novel step toward combining intentional canine behaviours with wearable technology, aligning with trends on the Internet of Medical Things. This proof-of-concept demonstrates technical feasibility and provides a foundation for future development of real-time seizure-alerting systems, representing an important first step toward scalable animal-assisted healthcare innovation.

## 1. Introduction

Epilepsy is one of the most common chronic neurological disorders, affecting an estimated 50 million people globally [1]. The condition is characterised by spontaneous recurrent seizures, which can lead to physical injury, psychological stress, and social isolation. The unpredictable nature of seizures poses risks for individuals with epilepsy as well as significantly limiting their quality of life [2].

Service dogs play a vital role in society by enhancing the safety, independence, and well-being of individuals through emotional and physical support, guiding those who are visually impaired, alerting to medical emergencies, and assisting people with PTSD or anxiety [3,4,5]. It is perhaps not surprising that assistance dogs have emerged as a supportive intervention, offering both physical and emotional aid prior to, during and after seizure events [6,7].

The human body secretes hundreds of different kinds of volatile organic compounds (VOCs) as part of our everyday activities, and these form a biomarker or fingerprint for each individual [8]. These VOCs are secreted from our breath, blood, and skin, and in our urine. Some VOCs are known to be biomarkers of infectious diseases and genetic disorders [9,10,11].

Studies on biomarkers linked to epileptic seizures have shown that VOCs unique to human seizures include Menthone which may be an important pre-ictal biomarker of impending seizure [12]. Scientists are still at the discovery stage for using VOCs and human scent for the elusive but desirable goal of seizure prediction. Research has found that the VOCs associated with pre-clinical seizures can be identified by canines with an accuracy of 82.2% [13]. Martos et al. [14] found that dogs displaying spontaneous seizure-alerting behaviour tended to have stronger emotional bonds with their owners and distinct personality traits, such as higher amiability and focus, compared to non-alerting dogs. Another study concluded that trained dogs could distinguish epileptic from non-epileptic seizures, reinforcing that VOC profiling holds promise for seizure detection [15]. These studies show the potential for seizure-scent-sensitised dogs as assistance dogs for early detection of seizure onset.

While previous reports have indicated that trained seizure-alert dogs can anticipate seizures and alert their handlers prior to onset [6,15], the findings of Powell et al. [16] are particularly noteworthy. In a controlled study, exposing 19 untrained pet dogs to sweat samples from individuals with epilepsy, they found that the dogs reliably exhibited affiliative behaviours, such as intense staring, pawing, and close contact, when presented with pre-seizure and seizure-phase odours. This suggests dogs can detect seizure-related volatile compounds. While promising, such behaviour is not yet fully understood, and scientific evaluations reveal inconsistencies in accuracy and reproducibility across individual animals [17]. In addition to this, a lack of objective, standardised tools for evaluating seizure-alert behaviours in dogs has limited both the validation and widespread deployment of such services.

Advancements in wearable sensor technologies and machine learning offer a compelling opportunity to enhance the capabilities of assistance dogs through automated seizure onset detection systems. While several wearable seizure monitors exist for human use show promise, most current devices either detect seizures only post-onset or suffer from false positives, low user-friendliness in assisted-living contexts, and lack of generalisability [18].

Substantial validation and personalisation are still needed to effectively integrate these devices into real-world support systems, such as those used alongside assistance dogs.

To date, there is little research into the use of seizure detection technologies designed for and worn by trained service animals. Recent work demonstrates the use of wearable accelerometers on trained assistance dogs to automatically detect signalling behaviours that predict impending seizures in humans, exemplifying early efforts to integrate seizure onsets [19]. Dogs were trained to alert (e.g., spin, jump, sit) on command and their movement data was logged directly by a movement sensor on their collar. Previous work by Raju et al. [19] demonstrated proof-of-concept behaviour recognition in assistance dogs using accelerometer-only data and within-dog cross-validation. While promising, this approach was limited by its reliance on per-dog models and susceptibility to sensor orientation drift. In the present study, we extend this work by introducing multimodal inertial sensing (accelerometer + gyroscope) using the Shimmer3 platform, which provides a richer motion representation and greater robustness to orientation. Methodologically, rather than training a separate classifier for each individual dog, we adopt a generalised model evaluated under a Leave-One-Dog-Out protocol, thereby testing performance on unseen dogs and improving the feasibility of deployment in real-world settings. Finally, whereas prior work evaluated performance at the level of whole events, we assess behaviours at a finer temporal resolution, allowing detection of shorter and faster signalling behaviours. Together, these advances address key limitations of earlier approaches and represent an essential step toward clinically viable seizure-alert assistance systems.

Building on these advances, the present study develops and validates a behaviour-alert detection collar designed for trained seizure-alert dogs. The collar integrates multimodal sensors and machine learning algorithms to detect seizure-associated behavioural changes and autonomously initiate a seizure alert. By incorporating real-time data analytics into an animal-worn device, this work demonstrates a non-invasive approach to enhancing seizure response systems. While prior studies have explored spontaneous seizure-alert behaviours in dogs, reliable, technology-assisted systems remain in their early stages. This proof-of-concept validation establishes the technical feasibility of wearable sensor technology for detecting trained signalling behaviours in assistance dogs and lays the groundwork for future real-world deployment.

The primary objective of this proof-of-concept study is to evaluate the collar’s detection accuracy for trained signalling behaviours under controlled conditions, establishing technical feasibility as a foundation for future naturalistic validation studies.

## 2. Materials and Methods

### 2.1. Overview and Research Setting

This study was funded by Research Ireland’s Frontiers for the Future Programme. conducted in partnership with Irish Dogs for the Disabled, a non-profit organisation specialising in the training of assistance dogs for individuals with physical and neurological disabilities, Epilepsy Ireland, the national charity for epilepsy in Ireland and Beaumont Hospital, a large teaching hospital located in Dublin, Ireland. The primary objective was to develop and validate a seizure-alert detection collar capable of recognising predetermined alerting behaviours in trained assistance dogs, as cued by trainers in this study, which in the future could be further evaluated for transmitting real-time alerts to owners, caregivers or emergency services.

This proof-of-concept study was designed to validate the core technical components of automated behaviour detection under controlled conditions. The controlled experimental environment was deliberately chosen to isolate technical variables and establish baseline system performance before progressing to more complex naturalistic validation studies.

### 2.2. Ethics Statement

This study was conducted in accordance with the ethical standards of the institutional research committees and in compliance with the Declaration of Helsinki and relevant animal welfare regulations. Ethical approval for research involving human participants was granted by the Beaumont Hospital Research Ethics Committee (Ref: 25/16) and by Dublin City University’s Research Ethics Committee (Ref: DCUREC/2025/065). All participants provided written informed consent prior to participation.

### 2.3. Device Design and Sensor Setup

The behaviour-alert detection system was implemented via a custom-designed smart collar that integrated the Shimmer3 Unit Sensor, an IMU sensor module featuring a 3D accelerometer and 3D gyroscope. The sensor was securely mounted on the collar to ensure the sensor was secure during all types of dog movement.

Sensor data was recorded locally on the device, not streamed in real time. Data was stored on an internal SD card (7.38 GB capacity), which enabled up to one week of continuous motion data collection which was time stamped to allow synchronisation with direct video recording of the training behaviours. In practical testing, a four-hour session recorded at over 50 samples/s required only 22.53 MB of storage, confirming the system’s efficiency for long-term deployment. The sensor was configured to sample at 50 samples/s, allowing for high-resolution capture of rapid motion events such as trained spin alerts as well as future experiments at lower sample rates by down-sampling. Power was supplied by a rechargeable battery with a tested lifespan of 8 to 12 h of continuous streaming operation. This ensured suitability for extended daily use without requiring frequent recharging.

After data collection, the sensor was physically connected to a Windows-based laptop via USB. The data were downloaded and processed using Consensys software v1.6.0 64-bit (Shimmer Sensing, Dublin, Ireland), which supported file conversion, time synchronisation, and raw signal extraction for analysis.

### 2.4. Training Seizure-Alert Behaviour

Six trained assistance dogs, Rosie, Stuart, Tori, Ranger, Nadia and Teddy participated in the study. All dogs were sourced from Dogs for the Disabled and were not privately owned. Dogs for the Disabled ethically breed and train Golden Retrievers, Labradors, and Standard Poodles, for disability assistance work. The dogs were all in good health and aged between 1 and a half and 2 years old. The dogs represented a range of common assistance breeds, including two Golden Retrievers, two Chocolate Labrador Retrievers, and two White Standard Poodles. Each dog had been previously conditioned by professional trainers to perform the spin alert behaviour upon command using shaping and positive reinforcement. The spin alert was defined as two rapid spins in a clockwise direction (as viewed from above), where the dog rotates its body in a full 360° circle, twice in immediate succession. Each rotation is completed within approximately 2 s, without interruption (e.g., no pausing, sitting, or changing direction between rotations). During observation periods, trainers elicited the spinning behaviour multiple times daily using controlled cues and documented each instance via timestamped video recordings. The use of trainer-cued behaviours in controlled settings is an appropriate methodology for proof-of-concept validation, allowing for precise behavioural standardisation and reliable ground truth labelling essential for initial algorithm development and validation.

The training and data collection protocol was designed to ensure consistency of the alert behaviour across dogs, naturalistic settings for behaviour performance and validated ground-truth labels through direct video observation.

### 2.5. Data Collection and Labelling

A comprehensive dataset was curated over a three-month period, resulting in the collection of 412.6 h of motion sensor data and 3078 s of synchronised video footage. The motion data was captured using an inertial measurement unit (IMU) on a smart collar, sampling at 50 Hz across six channels: three axes of linear acceleration (Accel_LN_X, Accel_LN_Y, Accel_LN_Z) and three axes of angular velocity (Gyro_X, Gyro_Y, Gyro_Z).

To establish ground truth for model training, a meticulous manual annotation process was undertaken by cross-referencing sensor data streams with the corresponding video recordings. The final annotated dataset comprised over 3000 s of time-series data, encompassing 135 distinct instances of the target spinning behaviour. These spin events accounted for a total of 349 s, with individual event durations ranging from 1.02 to 5.42 s. The data were collected from six canine subjects, exhibiting a notable imbalance in the distribution of events per subject: Stuart (*n* = 74), Rosie (*n* = 23), Teddy (*n* = 16), Ranger (*n* = 10), Tori (*n* = 9), and Nadia (*n* = 3).

**Figure 1:**
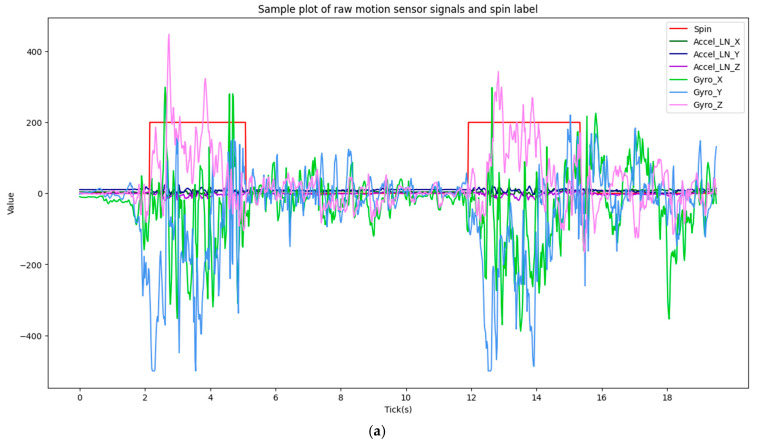

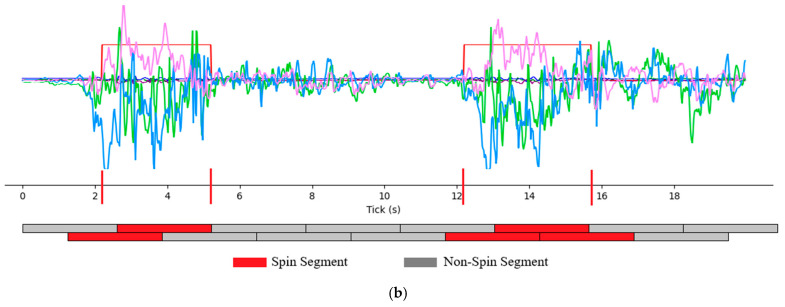
(**a**) Example of raw sensor data and annotation, (**b**) Data Segmentation with predefined window_size and stride_size. Graph represents accelerometer and gyroscope signals along the three IMU axes (X, Y, Z).

An example of the raw motion sensor signals recorded during a spin event is shown in Figure 1a, illustrating the characteristic periodic patterns that distinguish spinning from non-alert behaviours.

This labelled dataset represents a subset of the total 412.6 h of collected motion data; the remaining unlabelled portion was excluded from the current study but is reserved for future analysis.

### 2.6. Data Splitting and Segmentation Strategy

Raw time-series signals, such as shown in Figure 1a, are inherently continuous and variable in length, rendering them unsuitable for direct input into standard classification frameworks which typically require fixed-dimension feature vectors. To address this, a common and effective step in activity recognition is to segment the continuous data stream using a sliding window approach. This technique involves partitioning the data into short, overlapping segments of predefined *window_size* and *stride_size* (shown in Figure 1b). From each of these discrete temporal segments, a set of descriptive features (e.g., statistical, spectral) is extracted, transforming the time-series classification problem into a conventional supervised learning task where each window is represented by a single feature vector. These vectors serve as the fundamental input for developing either heuristic detection algorithms or training machine learning classifiers to predict the presence of the target behaviour.

**Figure 2:**
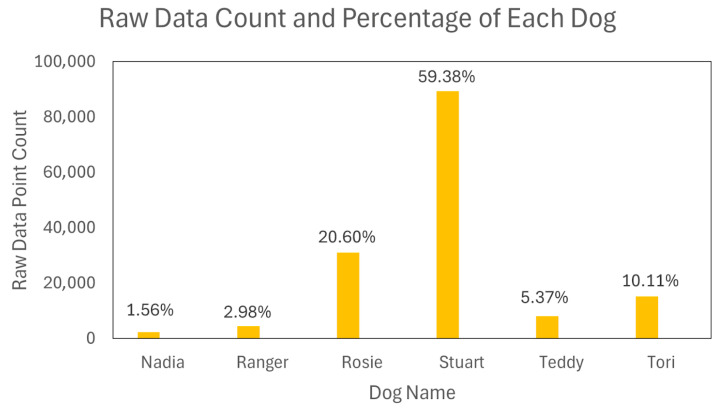
Raw data count and percentage for each dog.

A rigorous strategy was formulated for data partitioning and segmentation to ensure a robust and generalizable model evaluation. We employed a Leave-One-Dog-Out (LODO) methodology for the primary train-test split, designating the subject ‘Rosie’ as the hold-out test set. This choice provides a substantial and representative sample for evaluating generalisation, as Rosie’s data constitutes approximately 20% of the total dataset (Figure 2).

**Figure 3:**
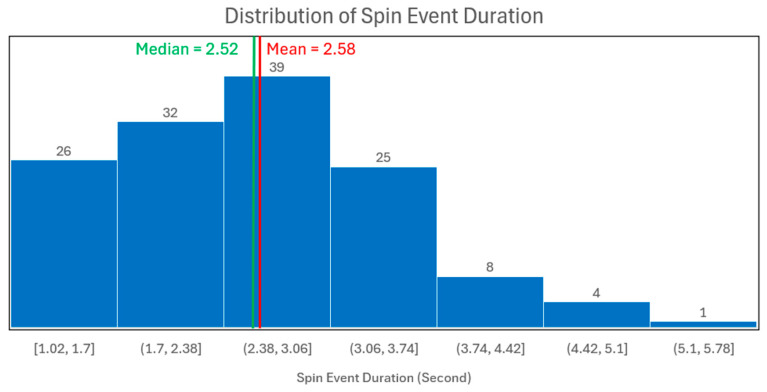
Distribution of spin event duration in second.

The time-series data was segmented using a sliding window approach, with parameters empirically derived from the event duration distribution (Figure 3). A 2.6 s window (window_size = 2.6 s), corresponding to 130 data points at 50 Hz, was selected to align with the mean (2.58 s) and median (2.52 s) event durations. A stride of 65 points (stride_size = 1.3 s) was used, creating a 50% overlap to augment the dataset. The label for each resulting segment was determined by its constituent points; if 50% or more of the data points within a window were part of a spin event, the entire segment was labelled as ‘spinning’, otherwise, it was considered ‘non-spinning’ (as in Figure 1b).

To ensure methodological rigour and prevent data leakage, the raw annotated dataset was first partitioned into a corpus of discrete events. A strict curation protocol was enforced whereby each event was defined to be behaviourally “pure”, ensuring it either contained one or more complete spin instances or was entirely devoid of any spin-related motion.

This collection of curated events was then used as the basis for all data splitting. For model training and evaluation, the events were partitioned into training and testing sets using a stratified 80/20 split. This event-based splitting methodology guarantees that no data from a single behavioural occurrence can span both sets, providing a valid assessment of the model’s generalisation performance.

### 2.7. Preprocessing and Feature Extraction

Feature extraction was performed directly on the raw, segmented time-series data from all six IMU channels. We opted to use the raw signals without attempting to separate gravity and body motion components, as the inconsistent orientation of the collar-mounted sensor makes such preprocessing unreliable. From each 2.6 s segment, an initial high-dimensional feature vector was engineered to capture both temporal and spectral characteristics of the motion signals, including statistical metrics (mean, standard deviation, range) and frequency-domain properties (dominant frequency, spectral energy, entropy).

To create an optimised and computationally efficient feature set for classification, this initial vector was refined using a three-stage feature selection pipeline:

- Low-Variance Filtering: Features with a variance below a threshold of 0.1 were removed to eliminate quasi-constant predictors.
- Collinearity Reduction: To reduce multicollinearity, a correlation analysis was performed, and one of any two features with a Pearson correlation coefficient greater than 0.95 was discarded.
- Univariate Feature Selection: Finally, an ANOVA F-test was employed via SelectKBest to identify the 20 features with the most discriminative power relative to the target classes.

This process converted each raw data segment into a final, optimised 20-dimension feature vector, providing a dense and informative representation of the underlying behaviour for the subsequent classification tasks.

### 2.8. Machine Learning Model Development and Evaluation

To establish a performance baseline and highlight the importance of our partitioning scheme, we also implemented a conventional within-subject scheme. This comparative approach eschews the LODO and event-based partitioning protocols. Instead, the continuous data streams from all subjects were first concatenated and then segmented using a sliding window. The complete corpus of resulting segments was subsequently subjected to a standard random train-test split to generate training and testing sets for model evaluation. While this method risks data leakage by allowing segments from the same subject or behavioural event to appear in both partitions, it provides a common reference point for model performance under less stringent generalisation constraints.

Adhering to the event-level partitioning and Leave-One-Dog-Out (LODO) protocol previously described, we developed and evaluated four supervised learning algorithms for the spinning detection task: Random Forest, Support Vector Machine (SVM), Naïve Bayes, and Logistic Regression. Besides these algorithms, we developed a heuristic baseline model to establish a clear performance benchmark. The baseline model classifies a segment as a “spin” if its mean gyroscope magnitude surpasses a specific threshold. To ensure a fair comparison with our supervised methods, this threshold was optimally tuned for each fold using only the training set data before being applied to the hold-out test set in both the within-subject and LODO evaluations.

The model’s efficacy was assessed using a dual framework to measure both granular classification accuracy and practical detection capability. At the segment level, performance was quantified using standard metrics, including the F1-score, accuracy, and ROC-AUC. However, given that a single 2.6s window may only capture a fragment of a behaviour, a more practical event-level evaluation was conducted to account for the high cost of missed detections (false negatives). For this, a dedicated test set of curated 7.8 s events from the held-out subject was used. An event was classified as ‘spin’ if at least one of its five constituent 2.6 s segments received a positive prediction, with final performance judged by the trade-off between missed detections and false alarms.

All model development was conducted in a Python (v3.10) environment using the scikit-learn (v1.7) and SciPy (v1.15.0) libraries.

## 3. Results

### 3.1. Comparison with Heuristic Methods

Table 1 presents a direct performance comparison between this heuristic baseline and the top-performing Random Forest model. While the heuristic method provides a reasonable baseline, the Random Forest model achieves a substantially higher F1-score and accuracy in both evaluation protocols. The performance gap is particularly pronounced in the more challenging LODO scenario, underscoring the superior generalisation capabilities of the data-driven supervised learning approach.

### 3.2. Supervised Model Performance

Having established the superiority of a supervised approach, we compared the four evaluated machine learning models. The comprehensive performance is summarised in Table 2.

The Random Forest model consistently demonstrated the most robust performance, achieving the highest scores across all metrics in both the within-subject and the more demanding LODO generalisation scenarios. An interesting trend emerged when comparing the evaluation protocols. While overall accuracy remained remarkably stable for all models when moving from within-subject to LODO evaluation, the F1-score showed a more distinct degradation. This suggests that the primary challenge in subject-independent generalisation lies not in overall classification correctness—which is heavily influenced by the majority ‘non-spin’ class—but specifically in the precise identification of the minority ‘spin’ class for unseen subjects.

### 3.3. Comparative Analysis of Segment- and Event-Level Performance

**Figure 4:**
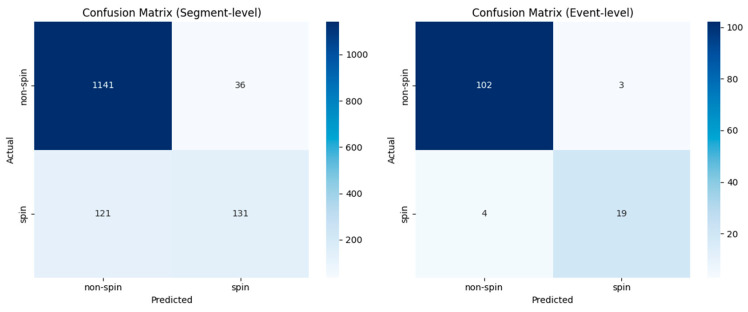
Confusion Matrices for Segment-Level and Event-Level Performance.

The performance of the Random Forest model was evaluated from two distinct perspectives: its raw predictive accuracy at the segment level under the LODO protocol, and its practical efficacy in a simulated event-detection scenario. This dual analysis provides a comprehensive understanding of both the model’s fundamental classification capabilities and its potential for real-world deployment. The confusion matrices for both evaluations are shown in Figure 4.

The segment-level performance was quantified by aggregating the predictions across all folds of the LODO cross-validation. This result reflects the model’s ability to generalise to unseen subjects on a moment-by-moment basis. The model correctly identified 1141 non-spin segments (true negatives) with only 36 false positives. However, while 131 spin segments were correctly classified (true positives), 121 were missed (false negatives). This indicates that although the model exhibits high specificity, its segment-level sensitivity is a significant limitation for an application where missed detections are critical.

To assess performance in a more realistic context, we simulated an event-detection task. For this, a test set of 7.8 s-long events were randomly extracted from our labelled data. The trained model generated predictions for each 2.6 s segment within these events, and these predictions were then aggregated to yield a single event-level classification. This event-level aggregation is designed to mirror a realistic scenario where a sustained behavioural episode, such as multiple consecutive spins, can be correctly identified even if the model produces intermittent, segment-level false negatives. This approach dramatically improved detection rates. The number of missed events (false negatives) was 4, with 19 spin events being correctly identified (true positives). This enhanced sensitivity came at the cost of 3 false alarms (false positives), while 102 non-spin events remained correctly classified (true negatives).

This comparative analysis demonstrates the transformative effect of temporal aggregation. The event-level simulation shows that by integrating predictions over a longer duration, the high false negative rate seen at the segment level is substantially mitigated. This trade-off, accepting a manageable increase in false alarms for a critical reduction in missed events, aligns perfectly with the primary goal of a reliable behaviour-alert system and underscores the necessity of using application-specific evaluation protocols to gauge a model’s true practical value.

## 4. Discussion

In human-worn wearables, machine learning methods (e.g., Logistic Regression, SVM, LSTM) have been successfully applied to seizure detection and forecasting, often using multimodal signals (EEG, accelerometry, ECG) [20,21]. While these studies demonstrate the power of advanced analytics, their designs, which rely on bodily biosignals, are intended for people with seizures to wear, rather than to include service animals. Our approach shifts the paradigm by capturing canine movement patterns as proxies for alerting behaviours, rather than relying on direct physiological measures of the human.

This proof-of-concept study demonstrates the technical feasibility of developing a robust behavioural detection system using wearable motion sensors and supervised machine learning. The findings show that a data-driven approach is decisively superior to heuristic baselines, with the Random Forest model consistently delivering the most robust performance, particularly in the challenging cross-subject generalisation protocol (LODO Accuracy = 0.92; F1-Score = 0.65). More importantly, this work highlights that practical reliability hinges on an event-level evaluation; by aggregating segment-level predictions over time, the system’s ability to avoid critical missed detections was dramatically improved, establishing a viable framework for a real-world canine behaviour-alert system.

Prior research has predominantly examined spontaneously occurring seizure-alert behaviours in pet dogs [17,22]. While these accounts suggest promising natural detection abilities, the findings often lack consistency, are based on owner-reported data, and have limited formal verification under controlled conditions. Our work builds upon that foundation by integrating structured data collection and machine learning pipelines, as exemplified in a recent study by Raju et al. [19], which implemented wearable accelerometers and classification algorithms to detect trained signal behaviours in assistance dogs, achieving similar performance metrics. These results support the feasibility of embedding intelligent behaviour recognition into assistance dog training programmes, potentially enhancing their reliability and scalability [20,23].

### 4.1. Robustness Across Canines

A primary objective of this study was to develop a model capable of generalising across different canines, a critical prerequisite for any real-world application. The Leave-One-Dog-Out (LODO) cross-validation protocol was specifically designed to test this capability, and the results confirm the robustness of the supervised learning approach.

The Random Forest model maintained high accuracy (0.92) and a strong F1-Score (0.65) even when evaluated on entirely unseen subjects. This performance stands in stark contrast to the heuristic baseline, where the performance gap was most pronounced in the LODO scenario, underscoring the necessity of a machine learning model to handle inter-subject variability in movement patterns, breed, and morphology.

Interestingly, the transition from within-subject to LODO evaluation revealed a key challenge. While overall accuracy remained stable across models, the more notable degradation in the F1-score suggests that the primary difficulty in generalisation lies in correctly identifying the minority ‘spin’ class for new individuals. This is further evidenced by the segment-level confusion matrix, which showed a significant number of false negatives (121) in the LODO protocol. However, our event-level analysis demonstrates that this challenge can be effectively mitigated. By aggregating predictions over time, the system successfully filtered out sporadic, segment-level errors, drastically reducing the number of missed events to a mere 6. This confirms that the proposed framework is not only capable of generalising across canines but is also resilient to the inherent variations in behavioural expression, making it a viable foundation for a widely deployable system.

### 4.2. Bridging Animal Behaviour and the Internet of Animals and Medical Things

While volatile organic compounds (VOCs) have been documented as potential pre-ictal biomarkers, with dogs capable of identifying them with up to 82% probability [13] technologies to monitor VOCs continuously are still developing, such as Sandia’s technical demonstration of a silicon “nose” detecting seizure gases 22 min pre-event [22]. Although direct olfactory monitoring remains promising, it is not yet wearable or field deployable. The wearable in this research offers an intermediate solution, combining proven canine scent detection [24] with wearable sensor technology and supervised signal processing. This aligns with broader trends in Internet-of-Medical-Things (IoMT) research, where wearable motion and physiological signals are increasingly analysed via machine learning for medical applications [23,25,26,27]. This system is positioned at the intersection of traditional animal-assisted interventions and emerging IoMT strategies, leveraging strengths from both domains.

Working assistance dogs occupy a distinctive role where human service and animal welfare meet. This necessitates an understanding of their subjective experience. Research in animal behaviour highlights the critical importance of positive human–animal interactions for promoting welfare outcomes in working dogs, suggesting that the quality of relationship between the handler and dog directly influences behavioural and emotional well-being [28]. Similarly, Lit et al. [29] demonstrated that handler beliefs can significantly affect scent detection performance, indicating that the human element plays a profound role in shaping operational efficacy and welfare. While chronic stress in dogs is detrimental, evidence suggests that an optimal level of arousal neither excessive nor insufficient is necessary to maintain engagement and prevent boredom, which itself poses a welfare risk [28].

The continued expansion of target odour repertoires has increased demand for scent detection dogs, placing growing moral and ethical responsibilities on those who work with and manage these animals. As Gandhi’s assertion that “the greatness of a nation and its moral progress can be judged by the way its animals are treated” gains renewed relevance, public scrutiny of animal use intensifies. In this context, adherence to transparent, evidence-based welfare practices is essential not only for ethical legitimacy but also for maintaining the social licence to operate. Science plays a pivotal role in this process, offering tools to both refine welfare protocols and critically reassess long standing practices in the light of evolving standards and expectations [29,30,31].

### 4.3. Implications for Real-World Deployment

The combination of a clear, intentional alert behaviour, actioned through wearable technology, fosters systematic integration with assistance dog training programmes. This addresses key concerns identified in seizure-alert dog literature regarding unpredictability and unverified alert behaviours. Moreover, the high performance of simple, interpretable models such as Logistic Regression suggests that complex deep learning pipelines, while powerful, may not be strictly necessary for behavioural event detection, especially when limited labelled data are available. This is consistent with broader observations in wearable IoMT literature, where simpler, explainable algorithms often outperform deep models when data are limited [26,32].

It is important to note that all training and interaction with the dogs adhered strictly to ethical, evidence-based positive reinforcement protocols, avoiding any use of aversive techniques. The dogs’ welfare and autonomy were paramount throughout. Each dog’s quality of life was monitored by experienced trainers. Key indicators such as engagement, stress-related behaviours, appetite, rest quality, and willingness to train were recorded and reviewed regularly. Further research into the deployment of the collars would ensure dogs continued to be treated ethically, and adjustments to training routines made as needed to support each dog’s well-being, ensuring that the system prioritises not only efficacy but also the dignity and welfare of the working animals involved.

### 4.4. Limitations

As a proof-of-concept study, this research has inherent limitations that define its scope and inform future research directions. These limitations are characteristic of foundational validation studies and provide clear guidance for subsequent development phases.

The dataset used for training and testing was relatively small, comprising 135 labelled events across six dogs, but this was an intentional design choice. In real-world deployment scenarios, where a behaviour-alert system would be tailored to an individual dog and person, the amount of available training data is inherently limited. This is because it is not practical for assistance dogs to perform excessive spin signalling during their own training. While this may be perceived as a limitation, it reflects a practical reality. Effective models must be able to learn from sparse, subject-specific data. For Ranger (*n* = 10) and Nadia (*n* = 3) the sample was particularly small. This data imbalance likely contributed to the reduced performance observed in certain folds of the LODO validation and underscores the need for more balanced datasets to enhance cross-subject generalisation.

A further methodological limitation lies in the interpretation of segment-level performance. While segment-level analysis is a standard approach for time-series classification, our results revealed its potential to be misleading for an event-based alerting application. The LODO cross-validation yielded 121 false negatives at the 2.6 s segment level, a figure that, in isolation, suggests a high risk of missed alerts. However, this granular view does not reflect the system’s true operational utility, as an alert is an event sustained over time, not a single-second classification. Our event-level simulation confirmed that by temporally aggregating these predictions, the vast majority of complete spinning events were successfully detected. This highlights a critical consideration for future work: a sole reliance on segment-level metrics can obscure the practical viability of an alert system and may lead to flawed conclusions about its deployment readiness.

While model performance was strong, further validation on larger and more diverse dog–handler populations is necessary to confirm generalisability. Additionally, the behaviours in this study were cued under controlled conditions by trainers, which may differ from spontaneous alerting responses observed in uncontrolled, real-world environments.

The use of a spin behaviour as the alert signal in this study was a deliberate, pragmatic choice aimed at reducing behavioural variability and enhancing experimental clarity. As spinning is both visually distinct and unlikely to occur spontaneously in a dog’s natural repertoire, it minimises false positives during trials. While effective for controlled research settings, spinning is neither a discrete nor universally appropriate behaviour for real-world environments, particularly in crowded or constrained spaces such as public transportation, where safety and subtlety are paramount, and spinning is not a natural reaction. This choice, though useful for validation purposes, inherently excludes other potentially more intuitive or organic alert behaviours like staring, barking, pawing, nudging or pacing, which some dogs may naturally use. Looking ahead, the system should be expanded to include a broader range of alert behaviours such as pawing, barking or sustained gaze. These behaviours may be more practical, discrete, and conducive to real-world application, allowing for both greater user adaptability and improved welfare considerations for the working dog [31].

It should also be noted that the alerts were elicited by professional trainers using controlled cues, rather than by the dogs’ independent detection of a pre-seizure event. While this may differ somewhat from real-world conditions, it still serves as a reasonable approximation. The controlled experimental conditions, while limiting immediate real-world applicability, represent a methodological strength for proof-of-concept validation. This approach allowed for precise isolation of technical variables, standardisation of behavioural signals, and establishment of robust ground truth data essential for algorithm development. These controlled conditions provide the necessary foundation for systematic progression to naturalistic validation studies.

### 4.5. Future Directions

Future research for our work will focus on scaling and refining the system to improve real-world readiness and clinical utility. This includes expanding the dataset across a larger number of dogs and simulated seizure contexts to improve generalisability and model robustness. Particular attention will be given to real-world deployment scenarios, where alerts would be triggered spontaneously prior to actual seizures, under varied environmental stressors and distractions. Controlled experiments with professional trainers offer consistency, but do not capture the full complexity of in-home, lived conditions. As such, validating the system in these naturalistic environments, including integration of real-time alert delivery through mobile app notifications or caregiver systems, will be essential. While the current model demonstrates strong detection accuracy, it does not assess latency or full end-to-end response performance. Future studies must test how quickly and reliably the system can deliver alerts to ensure timely intervention and improve user trust in urgent care scenarios.

Furthermore, a key aspect of this validation involves leveraging the extensive unlabelled portion of our dataset—over 400 h of motion data collected without concurrent video. A valuable future direction is to deploy the current model for inference across this entire corpus to quantify the baseline frequency of naturally occurring spin events. Such an analysis is critical for evaluating the ecological validity of using spinning as a distinct alert, distinguishing it from spontaneous, innate behaviours that may occur in a home environment.

Given the limitations of the current spinning behaviour as an alert signal, future versions will explore more natural and context-sensitive behaviours. Adaptive training models and customisable libraries of sensor-linked behaviours could help tailor alert signals to individual dogs, users, and environments. Expanding to multi-behaviour-alert repertoires not only improves ecological validity but also increases usability and safety in diverse real-world conditions. Ultimately, a system that is flexible, intelligent, and ethically aligned with both canine welfare and clinical needs will be key to transitioning from proof-of-concept to practice.

## 5. Conclusions

This proof-of-concept study successfully demonstrates the technical feasibility of using wearable sensor technology and machine learning to automatically detect trained alert behaviours in assistance dogs. Data collected from six dogs performing a standardised spin alert showed that the Random Forest model provided the most robust performance, achieving strong cross-dog generalisation in the Leave-One-Dog-Out evaluation (LODO Accuracy = 0.92; F1-Score = 0.65; ROC-AUC = 0.70). Although individual variability and limited data from some dogs presented challenges, the model’s accuracy remained consistently high across the cohort. Crucially, when evaluated at a practical event level, the system’s effectiveness was even more pronounced, drastically reducing the number of missed alerts compared to a simple segment-by-segment analysis.

This work represents an important step in developing technology-enhanced animal-assisted healthcare interventions. By combining the proven capabilities of seizure-alert dogs with objective sensor validation, this approach offers a pathway toward more reliable, scalable, and verifiable assistance animal services. As a foundational validation study, this research establishes the technical viability necessary for continued development of innovative seizure detection technologies that leverage the unique capabilities of trained assistance animals enhanced by modern sensor and computing technologies.

## Figures and Tables

**Table 1:** Performance comparison of the heuristic baseline vs. the best supervised learning model (Random Forest).

Model	Within-Subject Accuracy	Within-Subject F1-Score	LODO Accuracy
Heuristic Baseline	0.70	0.74	0.60
Random Forest	0.95	0.87	0.92

**Table 2:** Performance comparison of supervised learning models. The best-performing model across the most critical metrics is highlighted in bold.

Model	Within-Subject Accuracy	Within-Subject F1-Score	LODO Accuracy	LODO F1-Score	LODO ROC-AUC
Random Forest	**0.95**	**0.87**	**0.924**	**0.65**	0.70
SVM	0.94	0.83	0.920	0.54	0.59
Naïve Bayes	0.92	0.82	0.890	0.63	**0.77**
Logistic Regression	0.94	0.83	**0.924**	0.64	0.63

## Data Availability

Data supporting reporting findings can be found at https://figshare.com/articles/dataset/Package_of_motion_sensor_data_of_spin_alert_behaviour/30022441?file=57557335 (accessed on 1 September 2025).

## Abbreviations

The following abbreviations are used in this manuscript:

VOC/VOCs	Volatile Organic Compound(s)
IoMT	Internet of Medical Things
LODO	Leave-One-Dog-Out
SVM	Support Vector Machine
IMU	Inertial Measurement Unit
USB	Universal Serial Bus
SD	Secure Digital (card)
ROC-AUC	Receiver Operating Characteristic–Area Under the Curve
ECG	Electrocardiogram
EEG	Electroencephalogram
DCUREC	Dublin City University Research Ethics Committee
ANOVA	Analysis of Variance
